# Supplementary figures and images for: Whole-Genome Sequencing and Machine Learning Analysis of Staphylococcus aureus from Multiple Heterogeneous Sources in China Reveals Common Genetic Traits of Antimicrobial Resistance
Source: mSystems. 2021 Jun 8;6(3):e01185-20. doi: 10.1128/mSystems.01185-20 (PMC8579812; doi:10.1128/mSystems.01185-20)

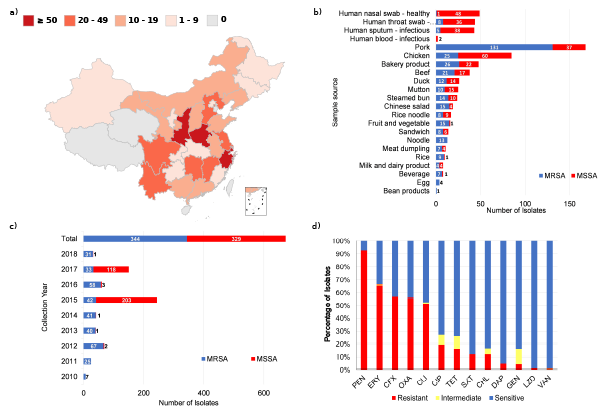

Supplement: FIG S1 [file msystems.01185-20-sf001.tif]

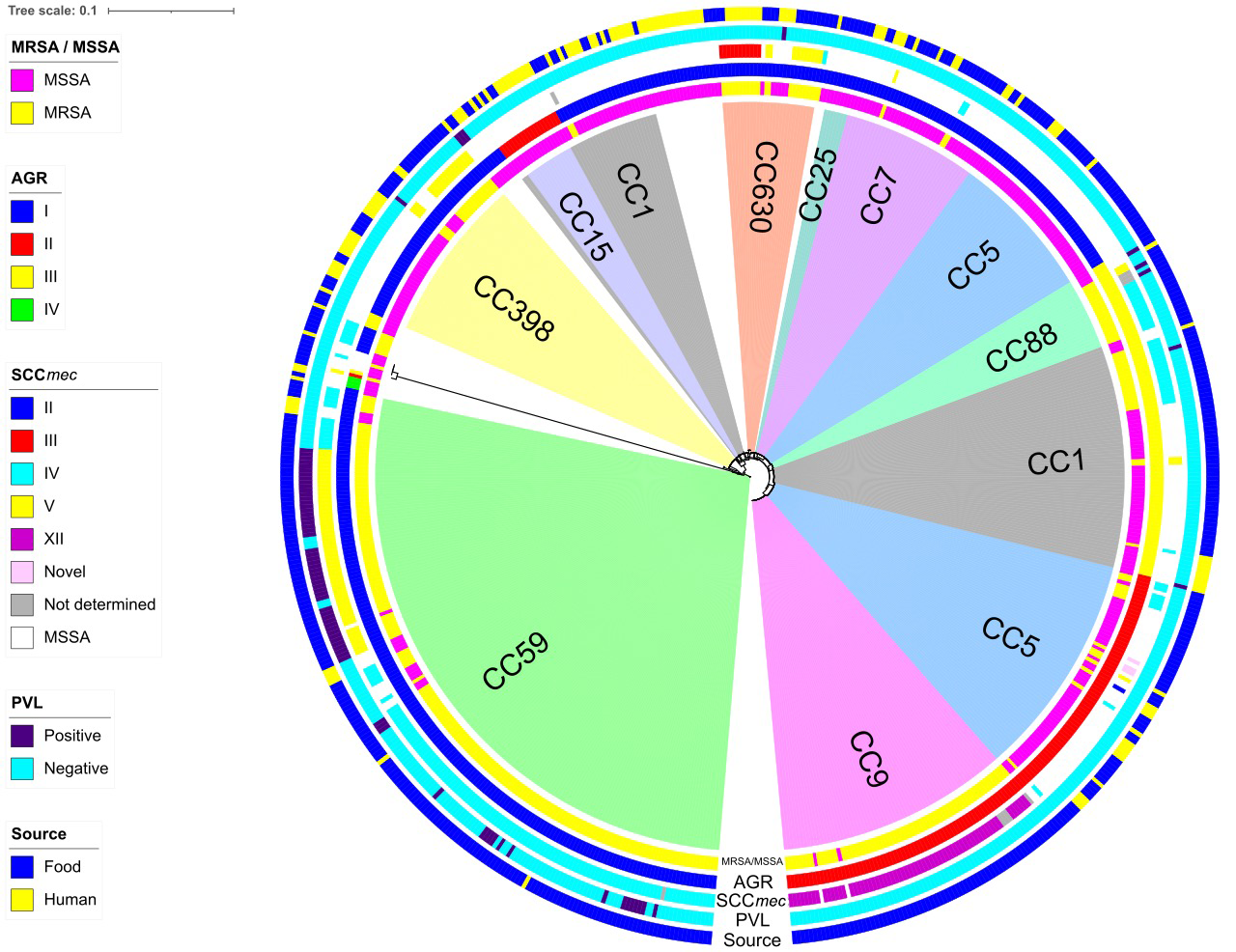

Supplement: FIG S2 [file msystems.01185-20-sf002.tif]
